# Gene expression profiling of peripheral blood cells for early detection of breast cancer

**DOI:** 10.1186/bcr2472

**Published:** 2010-01-15

**Authors:** Jørgen Aarøe, Torbjørn Lindahl, Vanessa Dumeaux, Solve Sæbø, Derek Tobin, Nina Hagen, Per Skaane, Anders Lönneborg, Praveen Sharma, Anne-Lise Børresen-Dale

**Affiliations:** 1 Department of Genetics, Institute for Cancer Research, Oslo University Hospital Radiumhospitalet, Montebello, Oslo, NO-0310, Norway; 2 DiaGenic ASA, Grenseveien 92, Oslo, NO-0663, Norway; 3 Institute of Community Medicine, University of Tromsø, Tromsø, NO-9037, Norway; 4 Department of Chemistry, Biotechnology, and Food Science, Norwegian University of Life Sciences, Ås, NO-1432, Norway; 5 Department of Radiology, Oslo University Hospital Ullevål, Oslo, NO-0407, Norway; 6 Institute of Clinical Medicine, University of Oslo, Oslo, NO-0316, Norway

## Abstract

**Introduction:**

Early detection of breast cancer is key to successful treatment and patient survival. We have previously reported the potential use of gene expression profiling of peripheral blood cells for early detection of breast cancer. The aim of the present study was to refine these findings using a larger sample size and a commercially available microarray platform.

**Methods:**

Blood samples were collected from 121 females referred for diagnostic mammography following an initial suspicious screening mammogram. Diagnostic work-up revealed that 67 of these women had breast cancer while 54 had no malignant disease. Additionally, nine samples from six healthy female controls were included. Gene expression analyses were conducted using high density oligonucleotide microarrays. Partial Least Squares Regression (PLSR) was used for model building while a leave-one-out (LOO) double cross validation approach was used to identify predictors and estimate their prediction efficiency.

**Results:**

A set of 738 probes that discriminated breast cancer and non-breast cancer samples was identified. By cross validation we achieved an estimated prediction accuracy of 79.5% with a sensitivity of 80.6% and a specificity of 78.3%. The genes deregulated in blood of breast cancer patients are related to functional processes such as defense response, translation, and various metabolic processes, such as lipid- and steroid metabolism.

**Conclusions:**

We have identified a gene signature in whole blood that classifies breast cancer patients and healthy women with good accuracy supporting our previous findings.

## Introduction

Cancer of the breast is the most common cancer among women worldwide with an estimated 1,300,000 new cases and 465,000 deaths annually [1]. In Norway, the age-adjusted incidence rate for breast cancer has more than doubled from 36.7 per 100,000 in the period 1953 to 1957 to 75.6 per 100,000 in the period 2003 to 2007 [2]. To reduce breast cancer mortality, early detection and appropriate treatment play a key role [3]. The five-year survival rate for stage I breast cancer in Norway in the period 1998 to 2002 was 95%, and 16.8% for stage IV metastatic breast cancer [2]. This emphasizes the importance of early detection so that treatment can be initiated as early as possible during tumor development. Mammographic screening, physical examination and self examination are the main modalities for breast cancer detection today, but only mammography screening has been shown to reduce mortality. When a tumor is detectable in the breast, either by palpation or mammography, the tumor might have been present for several years and have had the ability to spread to distant organs. The growth rate of breast tumors varies considerably between subjects [4]. Some tumors grow so rapidly that they escape a biannual screening program and hence show clinical symptoms before detection by mammography. In addition, mammographic sensitivity is significantly reduced in women with dense breast tissue, often seen in pre-menopausal women or those receiving menopausal hormone therapy [5]. Due to the low sensitivity of mammography in women with dense breast tissue, other imaging modalities have been introduced in breast cancer screening including ultrasonography and magnetic resonance imaging (MRI). However, ultrasound is very operator-dependent, time-consuming, and is associated with many false positive results. MRI is expensive, and the high false positive rate, limited resources and lack of universally accepted imaging guidelines restrict the use of MRI in a screening setting. The need for improved methods to accurately detect breast cancer at an early stage is highly desirable.

Previous studies have found that use of peripheral blood cells for transcriptome analysis is valuable to assess disease-associated [6-10] and drug-response related gene signatures [11]. We have previously demonstrated the potential use of gene expression profiling of peripheral blood cells for early detection of breast cancer [12]. Blood samples are easily available, minimally invasive and can be collected at low cost making them an attractive alternative modality for diagnostic purposes. The rationale for using blood as a clinical sample is that breast cancer triggers a response in circulating blood cells, leading to a traceable change in the whole blood gene expression signature. In this study we aimed to refine our previous findings [12] with a different sample set, using a larger sample size and a commercially available microarray platform.

## Materials and methods

### Subject information and blood sampling for microarray experiments

Two hundred blood samples were collected between 2002 and 2004 at two Norwegian hospitals (Ullevål University Hospital and Haukeland University Hospital) after written informed consent under approval from the Regional Ethical Committee of Norway (Ref. no. 416-01151). The subjects included were randomly selected among women called in for a second look after a first suspect screening mammogram. The samples were collected prior to a clinical examination that includes diagnostic mammography and biopsy or fine needle aspiration in the case of a positive mammographic finding. Cytology revealed whether the findings were of malignant or benign origin. For the subjects with no abnormal mammographic findings, the standard of truth was mammography alone. From each woman, 2.5 ml blood was collected in PAXgene™ tubes (PreAnalytiX, Hombrechtikon, Switzerland) and left overnight at room temperature before storing at -80°C until use. As a result of method development and testing of various gene expression platforms, only 121 of the 200 samples initially collected were included in this study. The diagnostic mammograms and histopathology reports revealed that out of these 121 women, 57 had invasive breast cancer, 10 had ductal carcinoma *in situ *(DCIS) and 54 had no sign of malignant disease. Of these latter 54, 12 had benign findings including fibroadenomas, cysts and some unspecified findings (Table 1). Regarding the breast cancer subjects, tumor stage, grade and other relevant clinical data were recorded (Tables 1 and 2). The individuals in the case and control groups are balanced in relation to age, menopausal status and previous menopausal hormone therapy (Table 3). In addition to the 121 samples, five blood samples were collected from two healthy women at multiple time points (biological replicates), three blood samples from pregnant women, and one sample from a breast feeding healthy woman were collected, leaving 130 samples from 127 individuals for gene expression analysis (Table 1).

**Table 1:** Clinical characteristics of the subjects included in the study (n = 127)

Diagnosis	Number of samples
**Total Breast Cancer**	**67**
**Pure DCIS**	**10**
Histological grade I	1
Histological grade II	2
Histological grade III	7
**Invasive Ductal Carcinoma (IDC)**	**49**
Histological grade I	11
Histological grade II	17
Histological grade III	16
Histological grade Unknown	5
**Invasive Lobular Carcinoma (ILC)**	**4**
Histological grade I	2
Histological grade II	2
Histological grade III	0
**Other invasive**	**4**
Invasive Tubular Carcinoma (ITC)	2
Medullary Carcinoma	1
Other/mixed cases	1
**Total Non-malignant**	63*
**Benign changes**	**12**
Fibroadenoma	1
Fibroadenoma and haematoma	1
Cyst	6
Unspecified findings	4
**No mammographic findings**	**42**
**Controls**	**9**
Breast feeding	1
Pregnant	3
Menstrual cycle (2 subjects)	5
**Total samples**	130*

* Data from biological replicates were merged leaving 127 assays for analyses.

DCIS = ductal carcinoma *in situ*

**Table 2:** ER and PR status among the 67 breast cancers samples

Status	Number of samples
ER+/PR+	36
ER-/PR-	7
ER+/PR-	7
ER-/PR+	1
Unknown	16

ER = estrogen receptor; PR = progesterone receptor

**Table 3:** Subject demographics

Demographic information	Breast Cancer N = 67	Healthy N = 60
**Age**		
Mean	58	56
Min	38	37
Max	82	70
Not registered	9	10
**Menopausal status**		
Pre-menopausal	14	15
Post Menopausal	37	29
Unknown	16	16
**Menopausal hormone therapy**		
Yes	13	13
No	54	57

### Study design

To control for technical variability such as different microarray production batches, lot variations of reagents and kits, day to day variations and effects related to different laboratory operators, a strict experimental design was followed. Samples were randomly divided into batches of 10, containing equal numbers of samples from women with breast cancer and those with no sign of the disease. All samples within each batch were handled together through each experimental step by one operator alone and the operators were blinded to cancer status. Two control samples were included in each batch following the same experimental procedures as the other 10. These control samples were composed of total RNA isolated from one healthy female. The order of the samples within each batch was randomized. In order to correct for any batch variations, we used the batch adjustment method described by Tibshirani [13]. A total of 13 batches including 130 samples and 26 technical controls were thus analyzed.

### RNA extraction

PAXgene™ tubes were thawed over night in batches of 12 tubes and total RNA was extracted according to the manufacturer's protocol. Total RNA was stored at -80°C prior to analyses. RNA quality and quantity measures were conducted using the 2100 Bioanalyzer (Agilent Technologies, Santa Clara, California, USA) and the NanoDrop ND-1000 spectrophotometer (Thermo Scientific, Wilmington, Delaware, USA) respectively.

### Microarray procedure

Microarray gene expression studies were conducted using single channel Applied Biosystems Human Genome Survey microarrays v2.0 containing 32,878 probes representing 29,098 genes. From each sample, 500 ng total RNA was amplified and labeled according to the NanoAmp RT-IVT Labeling Kit Protocol and hybridized onto the array for 16 hours at 55°C. Following hybridization, slides were manually washed and prepared according to the manufacturer's recommendation before image capturing using the AB1700 reader. Identification and quantification of gene expression signals, signal-to-noise ratios and flagging of failed spots were conducted using the Applied Biosystems Expression System software. Raw data files were exported for further analysis.

### Data analysis

Data analysis was performed using R [14] and tools from the Bioconductor project [15], adapted to our needs. Data was preprocessed in the following way: data were log2 transformed while individual measurements with signal-to-noise <3 or flag values >8,191 were set as missing. Probes with more than 5% missing values over all 156 arrays were excluded. Preprocessing left 156 samples and 11,217 probes for further analyses. Data were standardized (that is, centered and scaled) and missing values were imputed with k-nearest neighbors imputation [16] using k = 10. Principal components analysis and ANOVA tests for each gene revealed that there were large batch-effects present in the data. Similar batch effects have previously been reported for the same type of data (Dumeaux V, et al., under revision). Each probe was individually treated for batch effects using a one way ANOVA procedure as described by Tibshirani [13]. The 26 technical control samples were then excluded. For the biological replicates (multiple samples from one subject), signal intensities were averaged for each probe. Thus, 127 arrays, one from each individual remained for analysis. Finally, within-array normalization was conducted by global mean subtraction. The data discussed in this publication have been deposited in NCBI's Gene Expression Omnibus [17] and are accessible through GEO Series accession number [GEO:GSE16443].

### Feature selection and classifier construction

The gene expression data served as predictors for predicting a dummy-coded response vector. The response vector was given the value -1 or 1 for each sample depending on it being a healthy control or a breast cancer case, respectively. A new gene expression sample was classified as diseased if the predicted value was larger than zero and as healthy otherwise.

Partial Least Squares Regression (PLSR) [18,19] with double cross-validation was used to construct and test our classifier. PLSR with leave-one-out cross-validation (LOO-CV) was used in combination with Jackknife testing [20,21] to select significant probes. In more detail, LOO-CV gives the optimal number of components and a set of regression coefficients associated to each probe and jackknife feature selection is used to select probes with regression coefficients different from 0 (*P*-value ≤ 0.05). A PLSR model is rebuilt on these significant probes and LOO-CV is again used to select the optimal number of components. Finally, the analysis described above is incorporated in an independent loop of LOO-CV in order to test classifier accuracy [22].

### Functional enrichment analysis and biological interpretation

Reducing significant genes to core subsets is a useful step towards understanding biological mechanisms underlying the gene-set association with the phenotype of interest: a smaller number of genes are easier to understand and facilitate biological insight into disease processes. Global test [23] was used to identify the *core probes *most strongly explaining the difference between cases and controls. A Global test gene plot illustrates the influence of each individual probe on the significance result. The number of standard deviation of influence on the global test *P*-value above the reference line under the null hypothesis is termed the z-score. We identify probes with high z-scores (>2) as the core probes. Global test is not testing any specific null hypothesis. It is simply a useful analytical tool to reduce genes that have previously been found differentially expressed, to a core set, by gradually exploring the association of remaining genes as a set with a phenotype.

To explore functional enrichment and possible biological interactions among the genes identified we used the Database for Annotation, Visualization and Integrated Discovery (DAVID) [24], Human Experimental/Functional Mapper (HEFalMp) [25] and Graphle [26]. DAVID is a functional annotation tool able to extract biological information out of a large list of genes, while Graphle is an interactive tool displaying relationships between genes predicted by HEFalMp. HEFalMp predicts interactions between genes based on data integration of a vast number of experimental results publicly available and reduce all findings to a single measurement of relatedness [25]. Genes predicted to relate to each other often have a tendency to be co-regulated or are believed to carry out similar cellular tasks.

## Results

### Construction and characterization of the 738 classifier

Partial Least Squares Regression (PLSR) was used for model building while a leave-one-out cross validation (LOO-CV) was used to evaluate the use of PLS with LOO-CV and Jackknife testing for feature selection. We observed a high number of latent components necessary in the PLS model (N = 21) to achieve a cross validated minimum in the error rate.

**Figure 1:**
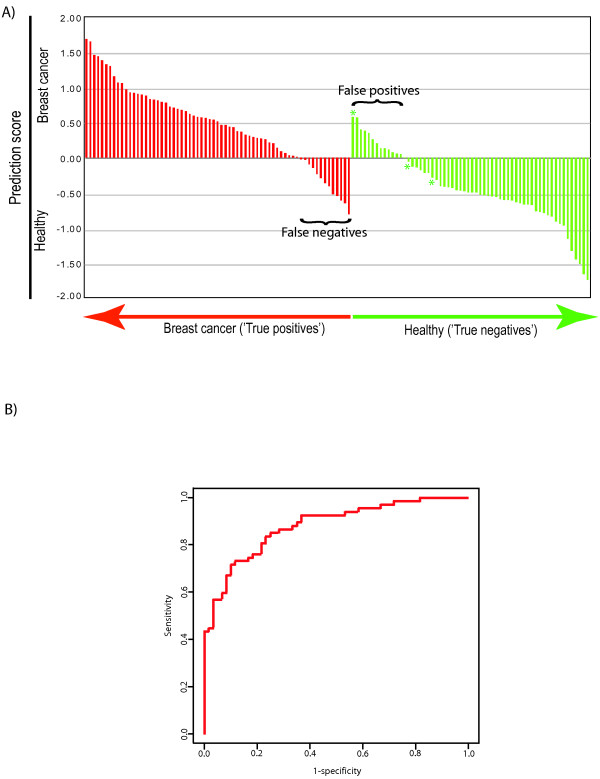
**Prediction performance**. **A) **Raw prediction scores (breast cancer >0> non-breast cancer). Subjects with breast cancer are indicated by red bars and healthy subjects are indicated by green bars. Among the 67 breast cancer patients, 54 were correctly predicted, while 47 of the 60 healthy samples were assigned to the correct class. False negatives (n = 13) and false positives (n = 13) can be seen in the centre of the figure. Bars marked with * are samples from pregnant women. **B) **ROC curve based on the double cross-validation results Prediction of the 127 samples based on the 738 probe list. The prediction accuracy is 79.5% and the area under curve (AUC) is 0.88 reflecting a good separation of the two groups.

By using PLSR with Jackknife testing on all samples (N = 127), a set of 738 probes were identified as significant for disease classification (*P *< 0.05) between breast cancer patients and women not having the disease. The 738 probe list predicted cases and controls with an estimated accuracy of 79.5% based on LOO-CV with a sensitivity of 80.6% and specificity of 78.3%. Of the 67 breast cancer samples, 54 were predicted correctly, while 47 of the 60 healthy samples were assigned to the correct class (Figure 1a). When plotting the sensitivity versus 1-specificity in a receiver operating characteristics (ROC) curve (Figure 1b), we observe a good separation of the two groups with an area under curve (AUC) of 0.88. Of note, a permutation test (k = 2,000) of the response variable gave a maximum accuracy of 60.6% and AUC of 0.68 (see Figures S1 and S2 in Additional file 1). To assess whether the results could be further improved by a larger sample size, random balanced subsets of samples were analyzed repeating the classifier building process. Results from the analyses indicate that accuracy could be increased with more samples. These results could in theory have been used to estimate a higher achievable AUC if more samples had been available. Attempts to model the results have however proven unsuccessful, consequently no upper AUC has been estimated (see Additional file 2). Using a Fisher exact test, we analyzed whether any of the clinical characteristics were significantly overrepresented among the subjects incorrectly predicted (Figure 1a). Of the false negatives, 11 out of 13 were samples from women having small lesions (<2 cm) including two DCIS, being significantly overrepresented with *P *= 0.04. Four out of 10 subjects with DCIS are incorrectly predicted as healthy, although not significantly (*P *= 0.09) overrepresented among the false negatives. Parameters such as tumor grade, estrogen receptor (ER) status or menopausal status do not seem to affect the prediction of the cases in this study. Samples from pregnant women or from women with benign lesions were not overrepresented among the false positives.

Global test gene plot (see Additional file 3) illustrates the influence of each individual probe in the 738 list on the significance result (*P *= 0.001). Approximately equal numbers of probes are up-regulated (n = 395) and down-regulated (n = 343) in blood of breast cancer patients, with the median z-score equal to 0.55 (sd 1.70) and 0.84 (sd 2.72) respectively. Z-score filtering (Z >2) left 89 core up-regulated probes and 119 core down-regulated probes. We used the core probes for gene interaction prediction.

### Functional enrichment analysis

Using DAVID [24] functional enrichment of the up- and down-regulated genes were investigated separately. Out of the 738 probes, a significant number were not annotated (n = 143) or had limited biological information and these were removed from the list along with duplicate gene symbols. Four hundred and ninety-three gene symbols from the total list were recognized by DAVID and included in the analysis. As background for the functional enrichment analyses, the 11,217 probes left after preprocessing were used. When analyzing the up-regulated genes alone we identified biological processes such as translation, defense response to bacterium, cellular biosynthetic process and response to external stimulus as enriched with false discovery rate (FDR) below 20% (Table 4), while processes involving various metabolic processes were enriched among the genes that were lower expressed in breast cancer patient compared to healthy controls (Table 5).

**Table 4:** Functional enrichment of genes expressed higher in blood of breast cancer patients compared to healthy subjects

Biological process	Count	%	*P*-value	Genes	Fold enrichment	FDR
**GO:0006412 Translation**	20	8.55%	0.0037	RPL26L1, LOC440587, RPS29, RPL37A, RPL11, UBA52, RPS3A, EEF1G, TRSPAP1, RPL36A, RPL24, RPL17, RPL14, RPL15, RPL4, RPL6, RPS25, ETF1, AARSD1, RPL12,	2.0	6.6
						
**GO:0042742 Defense response to bacterium**	5	2.14%	0.0064	DEFA3, LTF, CAMP, PPBP, S100A12,	6.5	11.3
						
**GO:0044249 Cellular biosynthetic process**	27	11.54%	0.0112	LOC440587, RPL26L1, ATP5E, UBA52, RPL11, RPL14, RPL4, ATP6V0B, RPS25, RPS29, RPL37A, RPS3A, ATP5L, EEF1G, TRSPAP1, RPL24, RNPEPL1, RPL36A, RPL17, GUK1, RPL15, PRODH, MTHFS, RPL6, ETF1, AARSD1, RPL12,	1.6	18.9
						
**GO:0009605 Response to external stimulus**	16	6.84%	0.0115	DEFA3, TIRAP, S100A12, CDKN2D, NMI, CXCR3, STAT3, RALBP1, CLU, PF4, AIF1, PPBP, C8B, CMTM5, ANXA1, GP1BB,	2.0	19.4

FDR = false discovery rate

**Table 5:** Functional enrichment of genes expressed lower in blood of breast cancer patients compared to healthy subjects

Biological process	Count	%	*P*-Value	Genes	Fold Enrichment	FDR
**GO:0044255 Cellular lipid metabolic process**	20	7.69%	0.0008	C10orf33, MBTPS1, PMVK, OSBPL7, SULT1A2, PEMT, LASS6, CMAS, SYK, PLAA, SULT1A4, INSIG1, IDI1, FDPS, HEXA, PECI, CYP2J2, ACAA1, SULT1A1, GRN,	2.3	1.6
**GO:0008202 Steroid metabolic process**	9	3.46%	0.0022	INSIG1, IDI1, MBTPS1, FDPS, PMVK, OSBPL7, SULT1A2, SULT1A1, SULT1A4,	3.8	4.0
**GO:0006629 Lipid metabolic process**	21	8.08%	0.0027	C10orf33, MBTPS1, ACAT2, PMVK, OSBPL7, SULT1A2, PEMT, LASS6, CMAS, SYK, PLAA, SULT1A4, INSIG1, IDI1, FDPS, HEXA, PECI, CYP2J2, ACAA1, SULT1A1, GRN,	2.1	4.9
**GO:0006584 Catecholamine metabolic process**	4	1.54%	0.0057	SULT1A2, HDC, SULT1A1, SULT1A4,	10.3	10.1
**GO:0018958 Phenol metabolic process**	4	1.54%	0.0057	SULT1A2, HDC, SULT1A1, SULT1A4,	10.3	10.1

FDR = false discovery rate

**Figure 2:**
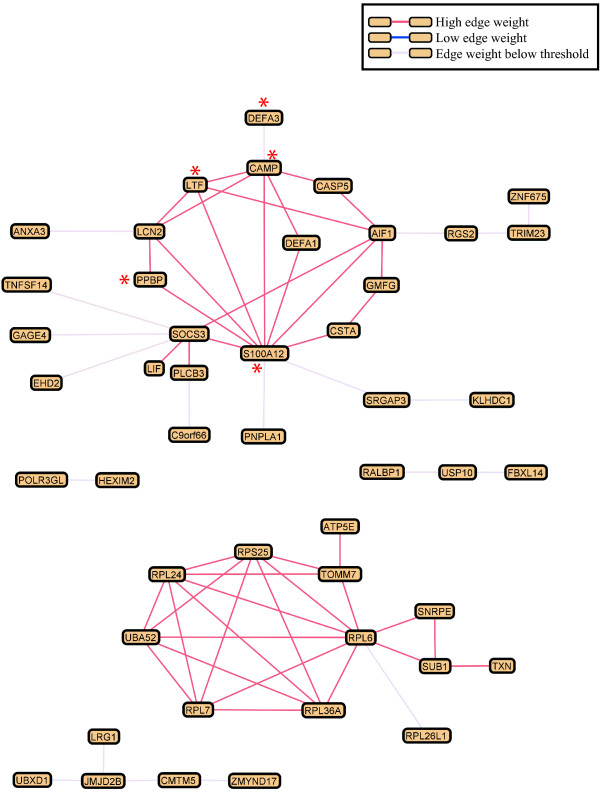
**Interaction map of the core up-regulated genes**. Biological network prediction of the 47 core up-regulated genes in blood of breast cancer patients compared to controls, using edge weight cutoff 0.648 (interaction confidence). Genes marked with red asterix are involved in defense response to bacterium.

Graphle/HEFalMp [25] was used to predict interactions between the genes within each group. When including hundreds of genes in such analyses, giant *hairballs *of predicted interactions are generated making the results hard to interpret. To reduce the complexity of the interaction maps we selected only the core genes (z-score >2) from the global test analysis (see Additional file 3). After removing probes without annotation, Graphle recognized 47 of the up-regulated core genes and 95 of the down-regulated core genes and predicted their interactions (Figure 2 and Additional file 4). Further, we submitted only the core genes to DAVID to look at functional enrichment within the core genes of each group in particular (see Additional file 5).

The interaction map for the 47 core up-regulated genes identifies two main networks and many of the genes within each network seem to be connected to each other with high interaction confidence (Figure 2). One cluster includes mainly genes coding for ribosomal proteins, playing different roles in the translation machinery. The other cluster contains among others, genes involved in defense response to bacterium. Ten genes are not connected to either of the clusters using edge filter cutoff 0.648 (interaction confidence). The 95 core down-regulated genes do not appear to be as strongly related to each other (see Additional file 4). We observe one main cluster with genes predicted to relate to each other with edge filter cutoff set to 0.643. Many genes cluster in small, more vague interaction networks. No biological processes were enriched among the 95 genes. Edge weights for the genes with highest relatedness are listed in Additional file 6.

Finally, we compared the 738 gene list to the 37 (29 unique) genes published in our previous study [12]. We applied the global test to our data to see whether the 37 gene set published in the initial study were differentially expressed between cases and controls. Twenty of the 29 unique genes were found in the filtered data of the present study, and this set of genes was not significantly differentially expressed between the cases and controls (see Additional file 7). Only two genes were overlapping between the two gene lists (*RPS2 *and *RPL14*), both coding for ribosomal proteins.

## Discussion

The biological signal from breast tumors recapitulated in whole blood does not appear to be very strong, reflected by the high number of latent components necessary in the PLS model. Other methods such as prediction analysis for microarray data (PAM) and support vector machines (SVM) were applied but did not improve classification accuracy (data not shown). Nonetheless, our results indicate that gene expression in whole blood serves as a possible diagnostic tool for early detection of breast cancer. We have identified a gene signature that separates breast cancer patients from healthy women with good accuracy. These results are in agreement with the findings in the pilot study, reporting a prediction accuracy of 82% [12] although for a different predictor. We use a rather liberal cut-off (*P*- value < 0.05) in the classifier construction and consider the probe list in biological terms, that is, several genes with moderate changes acting in concert within a pathway. The genes identified seem to reflect a biological response related to breast tumor growth. We also reduced the number of selected probes to a set of *core genes *more likely to be true positives and observe that similar biological processes are enrichment among the *core genes *up-regulated in blood of breast cancer patients.

### False negatives and false positives

The size of the mammary lesion is the only clinical feature that is significantly overrepresented among the falsely predicted samples. Lesions (including DCIS) with size below 2 cm were found significantly overrepresented among the false negatives. It is reasonable that a lower tumor burden will give a weaker response in blood affecting the prediction efficacy.

In our previous study all three pregnant subjects included were predicted as having breast cancer. In this study only one of the samples from the three pregnant women are predicted as having breast cancer.

Since mammography is the standard of truth, we can not exclude the possibility that some of the false positives have very early stage breast cancer or other occult tumors not detectable by existing technology. Follow-up data of these women are unavailable so we can not verify or falsify such a hypothesis.

### Biological interpretations

It is known that growing tumors communicate with the tissue in which they thrive, and also with the cells of the immune system of the host. The high rate of spontaneous occurring tumors in immunocompromised animals [27] and humans [28] reflects the inhibitory role of the immune system on tumor growth. The *blood-tumor dialogue *involves a broad spectrum of signaling molecules and such active cellular crosstalk seems to be reflected in the molecular blood signature of breast cancer patients discussed below.

A cancer-related gene expression signature in whole blood might reflect this communication. An increase or decrease of certain blood cell populations and their activities as a response to the tumor growth may also contribute to the observed difference.

Four biological processes are enriched with FDR below 20% when analyzing the genes up-regulated in blood of breast cancer patients (n = 243), including translation (GO:0006412), defense response to bacterium (GO:0042742), cellular biosynthetic process (GO:0044249) and response to external stimuli (GO:0009605). Among the genes down-regulated we identify processes involving lipid-, steroid-, catecholamine- and phenol metabolism (GO:0044255, 0008202, 0006629, 0006584, 0018958) as enriched.

Translation is a ribosome-mediated process where messenger RNAs (mRNAs) are translated into proteins. Translation is a process taking place in all cells, and it is difficult to draw any firm conclusions from this finding. However, in the pilot study we observed reduced expression of transcripts involved in protein synthesis among the breast cancer patients [12].

A defense related response observed in breast cancer patients is in agreement with our previous findings [12]. The five genes involved in defense response to bacterium are *DEFA3*, *LTF*, *CAMP*, *PPBP *and *S100A12*, genes that all are either highly expressed in neutrophil granulocytes or activators of such. Neutrophil granulocytes are the most abundant type of leukocytes (approximately 60%), whose role is to recognize and kill microorganisms, but also tumor cells [29]. Increased number of neutrophils (neutrophilia) is a sign of acute bacterial infection, but has also been reported in cancer patients, along with reduced lymphocyte counts (lymphocytopenia), referred to as an elevated neutrophil-lymphocyte ratio [30,31]. Whether such a shift in blood cell populations is due to defense related mechanisms or as a response to tumor derived signals is still not well understood. It has been proposed that tumor cells can attract neutrophils by secreting interleukin 8 (*IL8*) and that the neutrophils, in a similar manner as in wounds, enhance angiogenesis, tumor growth and progression, and finally cell migration through the ECM [32]. In contrast, one of the genes secreted by neutrophils; lactotransferrin (*LTF*) has been shown to have an inhibitory effect on tumor growth and metastasis via regulation of natural killer (NK) cell activity, modulation of expression of G1 proteins, inhibition of angiogenesis and enhancement of apoptosis [33,34]. Interestingly, the gene cystatin A (*CSTA*), a cystein proteinase inhibitor, which is among the 49 core up-regulated genes has been proposed as a prognostic marker for breast cancer [35,36]. Elevated lipocalin 2 *(LCN2*) levels has also been reported in tissue- and urine samples from patients with invasive breast cancer [37] and is proposed as a noninvasive biomarker for advanced breast cancer. It is believed that LCN2 promotes breast cancer progression by inducing epithelial to mesenchymal transition (EMT) and by increasing cell motility and invasiveness through down-regulation of E-cadherin.

Enrichment of genes involved in various metabolic processes among down-regulated genes suggests a change in the metabolism of breast cancer patients. Tumor growth often leads to dramatic metabolic changes in the host [38]. Several studies have shown altered systemic lipid metabolism in cancer patients [39], often leading to cachexia. Although cancer cachexia is most common in patients with terminal malignancies, it has also been observed in patients with a relatively small tumor burden [40]. The deregulation of lipid metabolism between cases and controls might reflect an early shift in the metabolism of the tumor bearer.

The gene interaction prediction analyses conducted using Graphle indicates that many of the core up-regulated genes seem to be linked to each other (Figure 2). When looking at the functional enrichment of the core up-regulated genes separately (see Additional file 5), we identify defense response to bacterium as the most significant process. This indicates that the core up-regulated genes carry much of the biological information that seems relevant in a blood-tumor dialogue context discussed above. We also identify taxis (GO:0042330, 0006935) as enriched among the core up-regulated genes alone. Taxis refers to movement of cells in response to external stimulus, possibly reflecting the movement of immune cells towards the growing tumor.

## Conclusions

The signature identified in this study is being further refined to improve the diagnostic accuracy. A TaqMan based clinical test, BCtect^® ^[41] has been developed in part based on the results from this study. This tool could constitute a fast and painless supplement to existing diagnostic technology, and offer a breast cancer test in areas where mammography screening is insufficient.

## Abbreviations

AUC: area under curve; CAMP: cathelicidin antimicrobial peptide; CSTA: cystatin A; DAVID: Database for Annotation, Visualization and Integrated Discovery; DCIS: ductal carcinoma in situ; DEFA3: Defensin, alpha 3, neutrophil-specific; ECM: extracellular matrix; EMT: epithelial to mesenchymal transition; ER: estrogen receptor; FDR: false discovery rate; HEFalMp: Human Experimental/Functional Mapper; IDC: invasive ductal carcinoma; IL8: interleukin 8; ILC: invasive lobular carcinoma; LOO-CV: leave-one-out cross-validation; LTF: lactotransferrin; LCN2: lipocalin 2; MRI: magnetic resonance imaging; mRNAs: messenger ribonucleic acids; NK cells: natural killer cells; PLSR: Partial Least Squares Regression; PAM: prediction analysis for microarray data; PPBP: pro-platelet basic protein (chemokine (C-X-C motif) ligand 7); PR: progesterone receptor; ROC: receiver operating characteristics; S100A12: S100 calcium binding protein A12; sd: standard deviation; SVM: support vector machines

## Competing interests

Torbjørn Lindahl, Derek Tobin, Nina Hagen, Anders Lönneborg and Praveen Sharma are employed by DiaGenic ASA and receive their salaries from the company. DiaGenic ASA develops diagnostic products for early detection of various diseases (including breast cancer). Anders Lönneborg and Praveen Sharma are co-founders of DiaGenic ASA and have substantial stocks in the company. Torbjørn Lindahl, Derek Tobin, Nina Hagen also have stocks/options in the company. Anders Lönneborg and Praveen Sharma are inventors of a gene-expression based method to detect disease, conditions or stages thereof (including breast cancer) using samples obtained from an area distant to the site of the disease (including peripheral blood). They protected the method by filing a patent in 1997. The company now holds several patents, and several applications are in process which combined covers the commercial use of the method and the products. The results disclosed in the present work are covered by DiaGenic's patent portfolio. The other authors declare that they have no competing interests.

## Authors' contributions

JA carried out the laboratory work (together with NH), participated in the discussion of the analyses, carried out the functional analyses, prepared the majority of the figures and wrote the manuscript. TL carried out the statistical analyses (together with SS) and wrote the manuscript sections concerning the statistical analyses. VD carried out the global test analysis, participated in the data pre-processing, prepared figures, participated in the discussion of the analyses and critical revision of the manuscript. SS carried out the statistical analyses (together with TL) and participated in the writing of the sections concerning the statistical analyses. DT participated in the discussion and critical revision of the manuscript. NH carried out the laboratory work (together with JA). PSk was responsible for collection of the blood samples, provided clinical information and critical revision of the manuscript. AL conceived and coordinated the study (together with PS and ALBD) and critical reading of the manuscript. PSh conceived and coordinated the study (together with AL and ALBD), participated in the discussion of the statistical analyses and critical reading of the manuscript. ALBD conceived and coordinated the study (together with AL and PS), and participated in the discussion of all analyses and critical revision of the manuscript.

## Supplementary Material

Additional file 1 Contains two figures, presenting the results from permutation tests (k = 2,000) of the response variables. Figure S1 shows a histogram of permuted accuracy values. The red line indicates the result presented in this study (79.5%) and is evidently better than that achieved by chance. Figure S2 shows a histogram of permuted AUC values. The red line indicates the result presented in this study (0.88) and is evidently better than that achieved by chance. Click here for file

Additional file 2 A figure presenting a learning curve - AUC improvement with increasing sample size. The figure shows the prediction accuracy with random balanced sample subsets, using an increasing number of samples and repeating the classifier building and testing process. The blue line indicates the mean AUC, while the light blue lines indicate one standard deviation from the mean. The red dot indicates result reported in this study. Extrapolation of the results does not indicate that the upper limit has been reached. The variance of the AUC decreases with higher percentages, this is an expected result from using more samples to validate the classifier. Click here for file

Additional file 3 A figure showing a ranked view of the 738 probes and their influence on the global test *P*-value. Probes with green bars show higher expression in blood of controls, while probes with red bars show higher expression in blood from women having breast cancer. The blue line indicates the influence of each probe on the global test *P*-value under the null hypothesis of no association. Black horizontal lines indicate one standard deviation of influence on the global test p-value above the reference line under the null hypothesis. The number of standard deviations is termed the z-score. Probes with high z-scores are the ones that most strongly explain the differences between cases and controls. The 208 core probes (z >2) are highlighted to the left. Click here for file

Additional file 4 A figure showing the biological network prediction of the 95 core down-regulated genes in blood of breast cancer patients compared to controls, using edge weight cutoff 0.643 (interaction confidence). Click here for file

Additional file 5 A table listing functional enrichment of core genes up-regulated in blood of breast cancer patients compared to healthy subjects. No biological processes were enriched among the 95 core genes down-regulated blood of breast cancer patients compared to healthy subjects. Click here for file

Additional file 6 A table listing the interaction confidence predicted by HEFalMp/Graphle between the core genes in each group (z-score >2). Click here for file

Additional file 7 A figure showing the influence of twenty of the annotated genes (some represented by multiple probes) from the 37 gene list published in the pilot study on the global test *P*-value in the present dataset. As illustrated by this plot, the enrichment of this set of 20 genes was not significant in relation to disease status in the present study. Only two of these genes are common with the 738 candidate gene identified; RPL14 and RPS2 (purple). Click here for file

Additional file 8 A recruitment overview of samples included in the study (n = 130). Click here for file
