# Supplementary figures and images for: Gene expression profiling of peripheral blood cells for early detection of breast cancer
Source: Breast Cancer Res. 2010 Jan 15;12(1):R7. doi: 10.1186/bcr2472 (PMC2880427; doi:10.1186/bcr2472)

S1

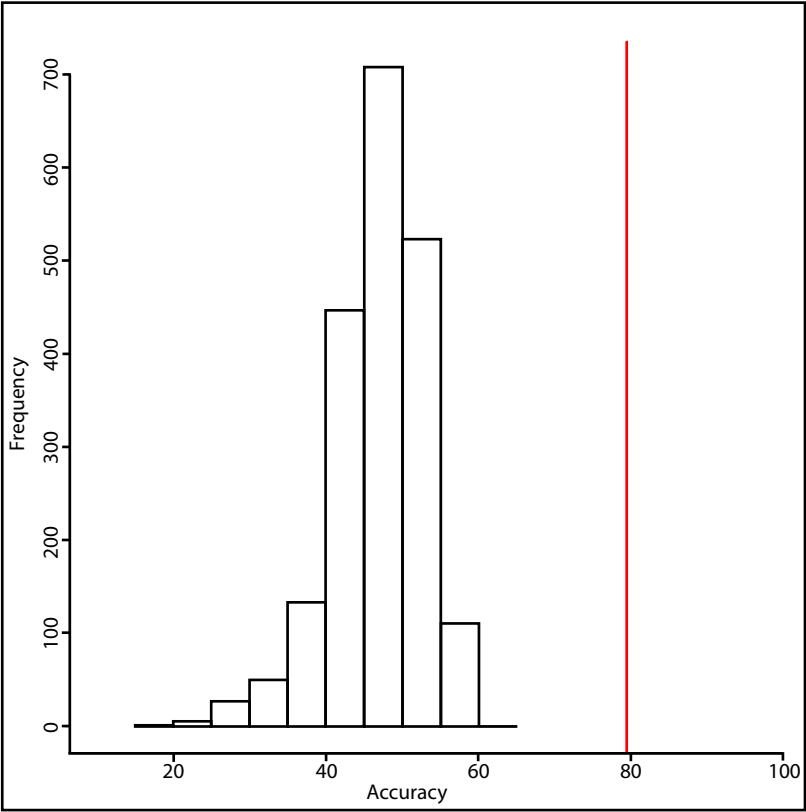

S2

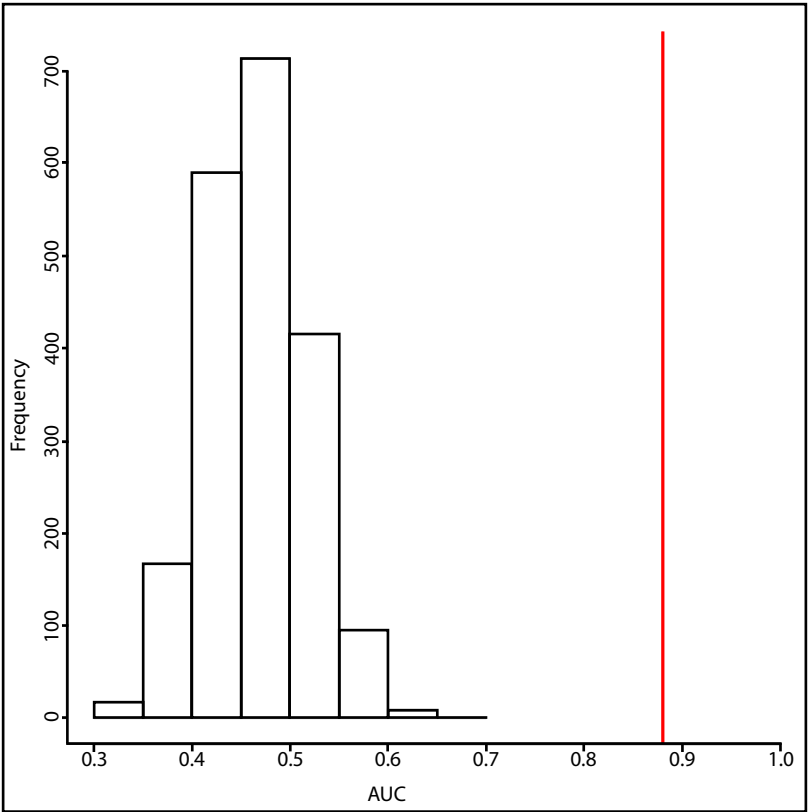

Supplement: Additional file 1 — Contains two figures, presenting the results from permutation tests (k = 2,000) of the response variables. Figure S1 shows a histogram of permuted accuracy values. The red line indicates the result presented in this study (79.5%) and is evidently better than that achieved by chance. Figure S2 shows a histogram of permuted AUC values. The red line indicates the result presented in this study (0.88) and is evidently better than that achieved by chance. [file bcr2472-S1.pdf]

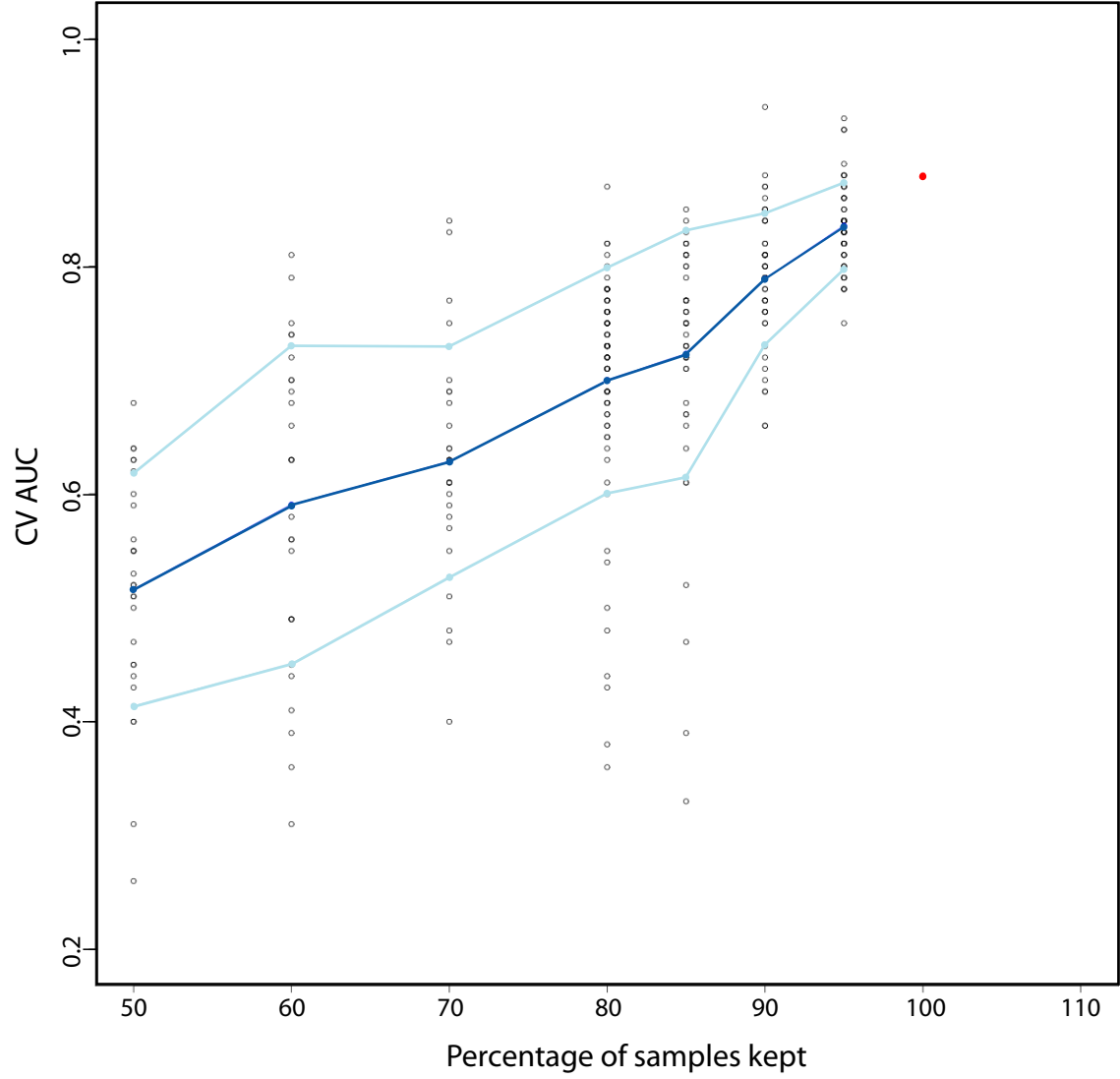

Supplement: Additional file 2 — A figure presenting a learning curve - AUC improvement with increasing sample size. The figure shows the prediction accuracy with random balanced sample subsets, using an increasing number of samples and repeating the classifier building and testing process. The blue line indicates the mean AUC, while the light blue lines indicate one standard deviation from the mean. The red dot indicates result reported in this study. Extrapolation of the results does not indicate that the upper limit has been reached. The variance of the AUC decreases with higher percentages, this is an expected result from using more samples to validate the classifier. [file bcr2472-S2.pdf]

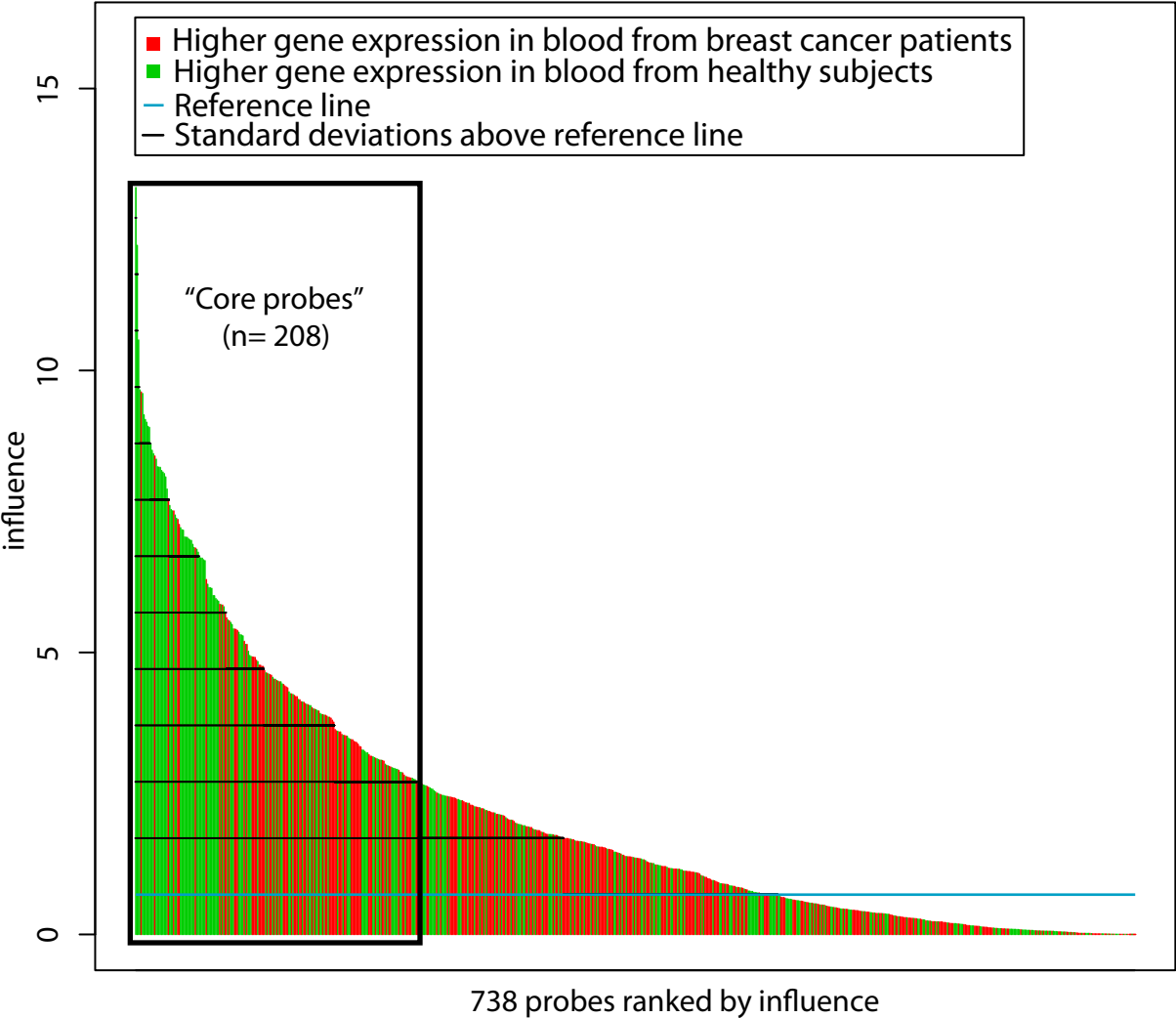

Supplement: Additional file 3 — A figure showing a ranked view of the 738 probes and their influence on the global test P-value. Probes with green bars show higher expression in blood of controls, while probes with red bars show higher expression in blood from women having breast cancer. The blue line indicates the influence of each probe on the global test P-value under the null hypothesis of no association. Black horizontal lines indicate one standard deviation of influence on the global test p-value above the reference line under the null hypothesis. The number of standard deviations is termed the z-score. Probes with high z-scores are the ones that most strongly explain the differences between cases and controls. The 208 core probes (z >2) are highlighted to the left. [file bcr2472-S3.pdf]

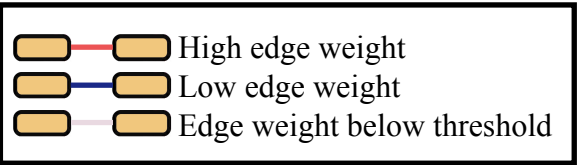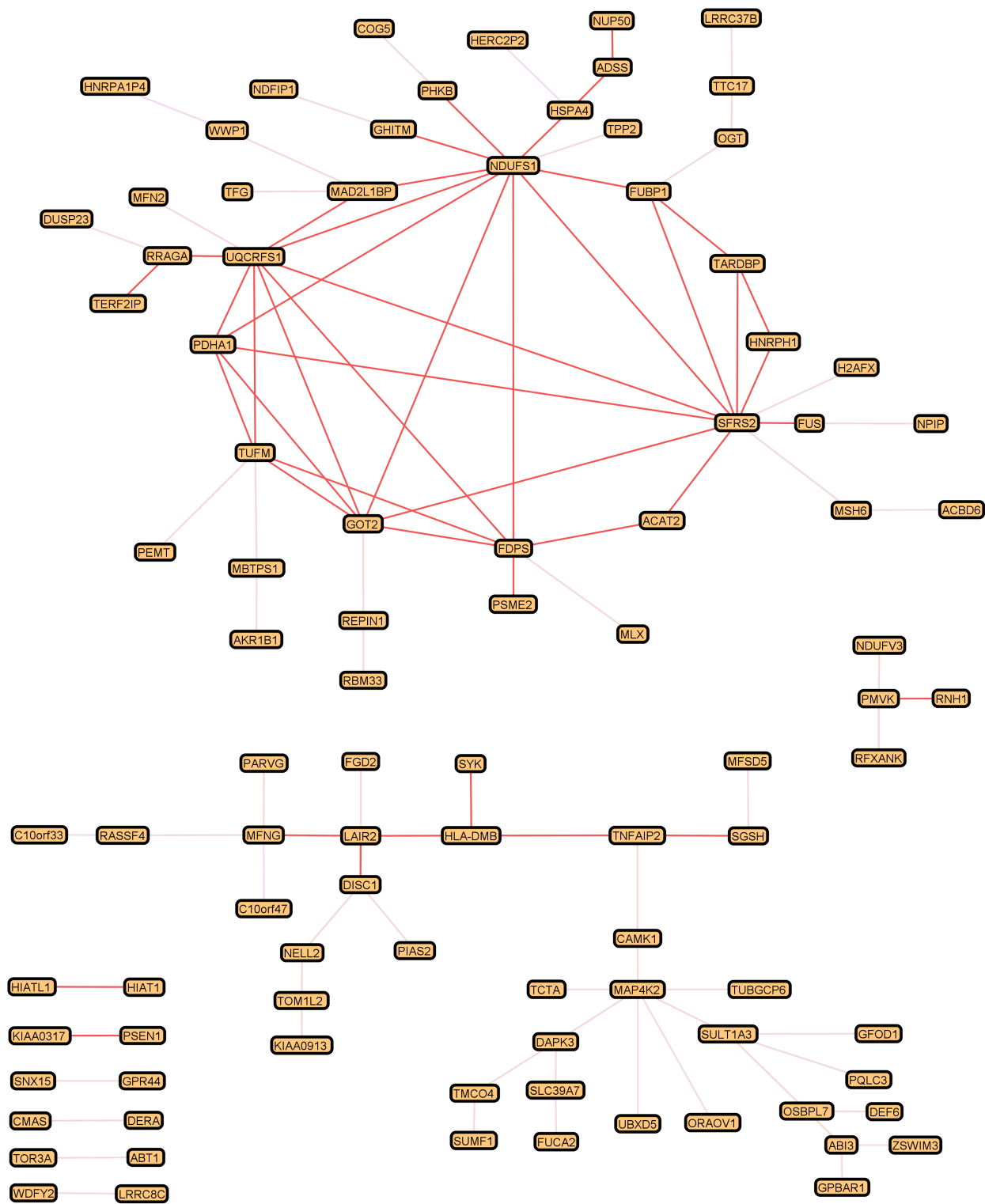

Supplement: Additional file 4 — A figure showing the biological network prediction of the 95 core down-regulated genes in blood of breast cancer patients compared to controls, using edge weight cutoff 0.643 (interaction confidence). [file bcr2472-S4.pdf]

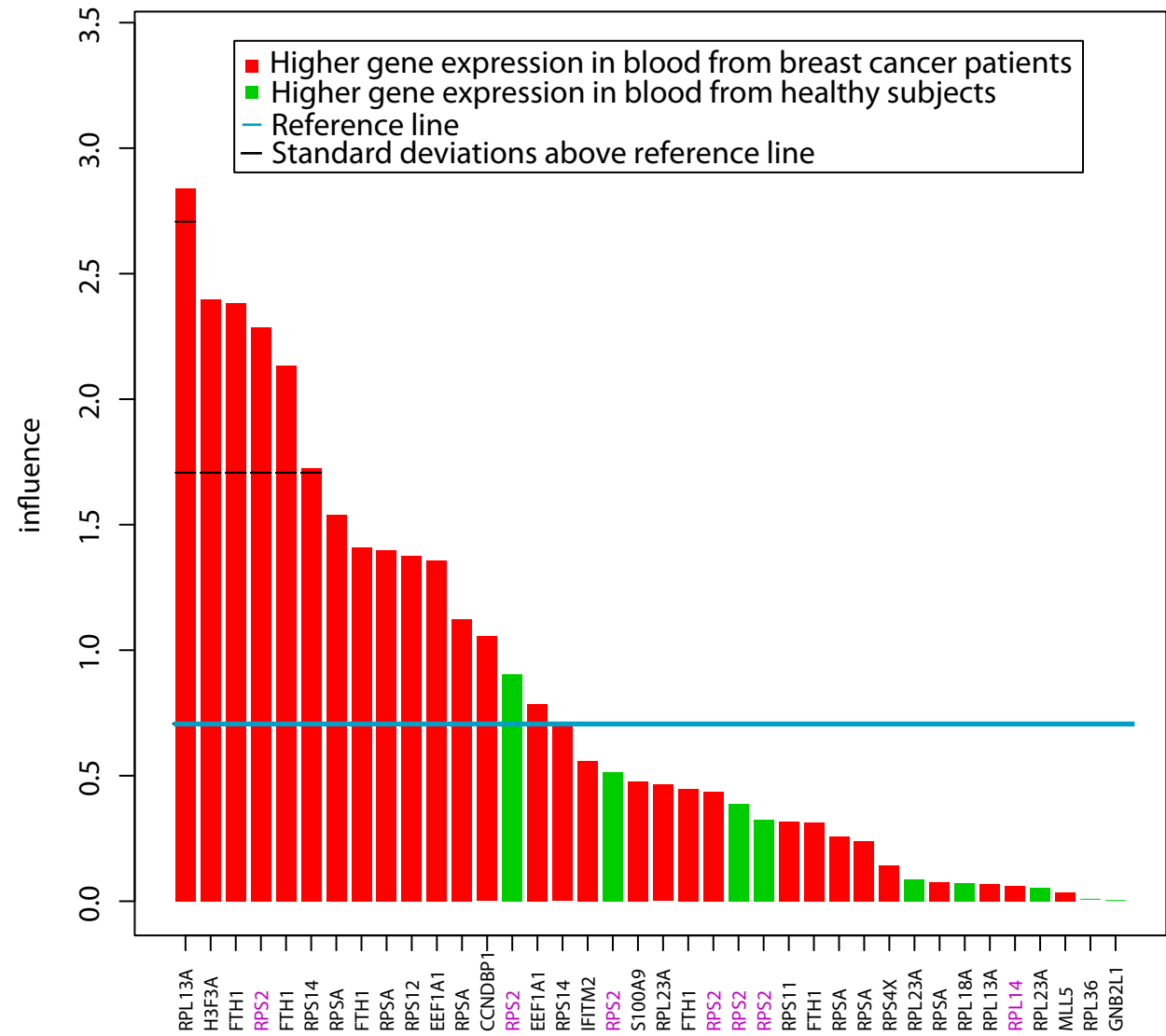

Supplement: Additional file 7 — A figure showing the influence of twenty of the annotated genes (some represented by multiple probes) from the 37 gene list published in the pilot study on the global test P-value in the present dataset. As illustrated by this plot, the enrichment of this set of 20 genes was not significant in relation to disease status in the present study. Only two of these genes are common with the 738 candidate gene identified; RPL14 and RPS2 (purple). [file bcr2472-S7.pdf]
